# Editorial: Cystic kidney diseases in children and adults: from diagnosis to etiology and back

**DOI:** 10.3389/fped.2024.1401593

**Published:** 2024-04-10

**Authors:** Lovro Lamot, Ivana Vuković Brinar, Margareta Fištrek Prlić, Bodo Beck

**Affiliations:** ^1^ University of Zagreb School of Medicine, Zagreb, Croatia; ^2^ Division of Nephrology, Dialysis and Transplantation, Department of Pediatrics, University Hospital Center Zagreb, Zagreb, Croatia; ^3^ Department of Nephrology, Arterial Hypertension, Dialysis and Transplantation, University Hospital Center Zagreb, Zagreb, Croatia; ^4^ Institute of Human Genetics, Center for Molecular Medicine Cologne, and Center for Rare and Hereditary Kidney Disease, Cologne, University Hospital of Cologne, Cologne, Germany

**Keywords:** cystic kidney disease, autosomal dominant polycystic kidney disease (ADPKD), autosomal recessive polycystic kidney disease (ARPKD), nephronophtisis complex (NPHC), Bardet Biedl syndrome (BBS)

**Editorial on the Research Topic** Cystic kidney diseases in children and adults: from diagnosis to etiology and back

**Figure 1:**
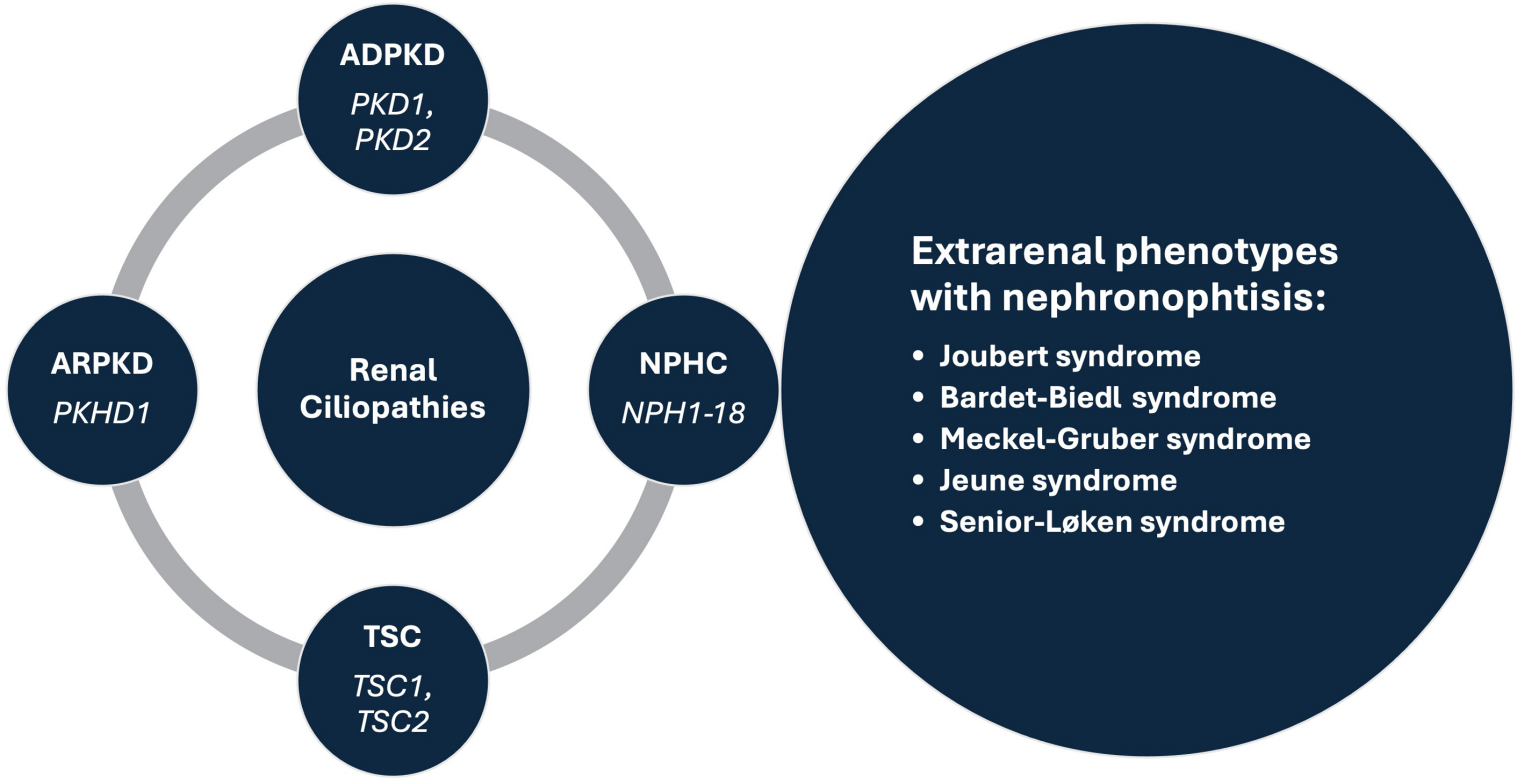
Prominent syndromes and associated genes within the renal ciliopathies concept. ADPKD, autosomal dominant polycystic kidney disease; ARPKD, autosomal recessive polycystic kidney disease; NPHC, nephronophthisis complex; TSC, tuberous sclerosis complex.

Renal cysts are often regarded as the most common abnormality associated with kidney disease (1, 2). They are encountered in both adults and children, as isolated findings or as part of a more complex clinical condition (3–5). Isolated kidney cysts in adults sometimes require evaluation for kidney cancer or simple cysts may occur as a sign of age-related kidney tissue degeneration in the absence of any underlying specific kidney disease. Recent advances in understanding the underlying mechanisms have led to the concept of renal ciliopathies with more than 100 genes associated with ciliary dysfunction, resulting in conditions such as polycystic kidney disease (PKD), tuberous sclerosis complex (TSC) and nephronophthisis complex (NPHC), which may be associated with various extrarenal phenotypes (Figure 1) (6–8). In addition to progressive CKD, these disorders are characterized by a variety of additional symptoms such as hepatic impairment, vision problems, developmental delays, intellectual disabilities, and skeletal abnormalities, which inconsistently present throughout the course of the disease (4, 5, 7). Furthermore, the significant phenotypic overlap makes it difficult to differentiate specific disorders, often necessitating genetic testing to reach a definite diagnosis (9). Despite a multitude of clinical and translational studies, in the majority of cases it is still challenging or even impossible to predict the individual clinical course, necessitating regular follow-up of the patients and a timely response in terms of treatment, which remains mostly symptomatic (10).

The present special issue contains seven noteworthy articles describing engaging cases of children and adults with various disorders having a common denominator in the form of kidney cysts, systematically reviewing the current literature on the clinical characteristics of an *HNF1B* gene variant and biomarkers of kidney disease progression in autosomal dominant PKD (ADPKD), investigating the outcome of fetal renal cystic disease and exploring the utility of magnetic resonance imaging-based kidney volume assessment for risk stratification in children with ADPKD.

In more detail, Simičić Majce et al. describe a nonconsanguineous family with three members affected by BBS caused by compound heterozygous mutations in the *BBS12* gene. Despite identical genotypes, the affected family members demonstrated significant diversity in clinical characteristics (different expressivity) of the BBS phenotype emphasizing the importance of genetic testing for the early diagnosis of this rare ciliopathy. Similarly, Fištrek Prlić et al. present two clinically distinct cases of autosomal dominant tubulointerstitial kidney disease (ADTKD) diagnosed only after genetic testing, along with an extensive review of the literature and a comprehensive overview of the condition. Both patients had uninformative renal ultrasound and urinalysis findings with only elevated serum creatinine levels indicating a kidney disease. An adult patient with a positive family history of CKD had no other symptoms, while an adolescent boy with an unremarkable family history had psychomotor impairment with epilepsy. After the testing they were diagnosed with *MUC1*-related ADTKD and 17q12 microdeletion syndrome causing the loss of one copy of the transcription factor *HNF1B* and 14 additional genes, respectively, highlighting the importance of clinical awareness in diagnosing this syndrome. Finally, the third case report by Kasahara et al. advocates an interesting option to treat the chronic pain experienced by more than half of patients with ADPKD. They describe an adolescent girl with persistent pain associated with multiple renal cysts that prevented her from participating in daily activities. After being diagnosed with attention deficit disorder (ADHD) and appropriate treatment for this condition being initiated, she experienced significant pain relief and better control of her hypertension. Therefore, in patients with ADPKD it may be important to recognize concomitant ADHD and consider a trial of ADHD medications when chronic pain associated with ADPKD is present.

In line with the exploration of important associations between cystic kidney disease and other disorders a systematic review by Nittel et al. examined the prevalence of neurodevelopmental disorders (NDD) in patients with 17q12 microdeletions vs. *HNF1B* point mutations. The results of a diligent literature search revealed that NDDs are frequently observed in *HNF1B*-associated diseases, especially in the common 17q12 microdeletion, and should hence become a routine part of clinical care for patients with *HNF1B*-related diseases. On the other hand, a systematic review by Sorić Hosman et al. provided a critical overview of previously examined serum and urine biomarkers with a potential for predicting disease progression or response to therapy in patients with ADPKD. A comprehensive literature review identified several prognostic molecules that are involved in various processes central to the development of the disease, such as tubular injury, inflammation, metabolism, renin-angiotensin, or vasopressin system adjustments. Interestingly, the most accurate predictive models have been achieved when incorporating such serum and urine biomarkers with the Predicting Renal Outcome in Polycystic Kidney Disease (PROPKD) score which combines underlying genetic mutations and clinical risk factors, or with the Mayo Imaging Classification (MIC) which is based on age- and height-adjusted total kidney volume (TKV) measured by magnetic resonance imaging (MRI).

MRI-based kidney volume assessment was further investigated by Yilmaz et al. in a multicenter, cross-sectional, and case-controlled study involving 89 children and adolescents with a genetically confirmed and detailly characterized diagnosis of ADPKD. The study patients were stratified according to the innovative Leuven Imaging Classification (LIC) into different risk categories, with those in the highest risk category having an increased incidence of hypertension and a higher prevalence of *PKD1* mutations. Therefore, the study advocates the use of MRI for the measurement of TKV in the pediatric population, in addition to the use of ambulatory blood pressure monitoring to recognize those with hypertension.

Finally, Botero-Calderon et al. presented a retrospective study evaluating clinical and imaging data, genetic testing results and postnatal follow-up outcomes of infants identified *in utero* with bilateral renal cystic disease at a single referral center over a period of 11 years. Among 17 patients with suspected renal ciliopathy, the most common diagnosis was autosomal recessive PKD (ARPKD, *n* = 4), followed by Bardet-Biedl syndrome (BBS, *n* = 3), autosomal dominant polycystic disease (ADPKD, *n* = 2), HNF1B-related disease (*n* = 2), and Meckel-Gruber syndrome (MKS, *n* = 2), while four cases were not genetically resolved. In terms of postnatal management, the study revealed that the vast majority of neonatal survivors with renal ciliopathies are directed to the care of a pediatric nephrologist, while this proportion is much lower in those with genetically unresolved enlarged, echogenic kidneys, stressing the need for structured management programs for prenatally identified kidney disease.

In conclusion, our research topic provides a contemporary overview of current practices, unmet clinical needs and research gaps regarding the broad spectrum  of renal ciliopathies that may be useful to a wide range of physicians and researchers dealing with these complex disorders.
